# Modulation of Brain‐Kidney Crosstalk by Olanzapine in Aluminum Chloride‐Induced Memory Impairment in Male Mice

**DOI:** 10.1002/alz70859_105486

**Published:** 2025-12-26

**Authors:** Lawrence D Adedayo

**Affiliations:** ^1^ Federal University Wukari, Wukari, Taraba Nigeria

## Abstract

**Background:**

Chronic kidney disease (CKD) is associated with an increased risk of neuropsychiatric disorders, cognitive decline, dementia due to the release of cytokines and chemokines, production of reactive oxygen species, and the generation of trophic factors. The second‐generation antipsychotic olanzapine (OLN) has been studied for its neuroprotective and antioxidant properties that may enhance cognitive function. However, limited research has explored the interaction between brain cytokines and kidney biomarkers, which could contribute to pathological conditions. This study aimed to assess the effects of OLN on cognitive function, oxidative stress, and the relationship between brain and kidney markers in mice with aluminum chloride (AlCl₃)‐induced memory impairment.

**Method:**

Twenty‐five male mice (20‐30 g) were randomly assigned to five groups (*n* = 5 each). Group 1 served as the control, receiving only distilled water (10 ml/kg). Group 2 received AlCl₃ (100 mg/kg). Groups 3 and 4 were pre‐treated with OLN (0.5 mg/kg and 1.0 mg/kg, respectively) one hour prior to AlCl₃ administration. Group 5 was pre‐treated with Donepezil (1 mg/kg). All treatments were administered orally for seven consecutive days. On the final day, neurobehavioral tests, including the Y maze and open field tests, were conducted to evaluate memory impairment. The mice were then euthanized; their brain and kidney tissues were analyzed for tumor necrosis factor‐alpha (TNF‐α), antioxidant markers, urea, and total protein levels, along with histopathological examination of the hippocampus and prefrontal cortex.

**Result:**

Both doses of OLN significantly improved cognitive function in the Y maze and open field tests, reduced oxidative stress by decreasing malondialdehyde and increasing SOD, catalase, and glutathione levels, and lowered brain TNF‐α levels. Histopathological assessments indicated that OLN reduced neurodegeneration in the hippocampus and prefrontal cortex. There is also decreased kidney urea and increased protein concentration.

**Conclusion:**

OLN significantly improves memory and antioxidant levels while reducing cytokine levels, although its effects on kidney function are limited. These results underscore the intricate relationship between OLN, cognitive function, and renal health.

